# The practice of integrated healthcare and the experiences of people in Ghana’s Ashanti region

**DOI:** 10.1186/s12913-021-07340-0

**Published:** 2022-01-05

**Authors:** Irene G. Ampomah, Bunmi S. Malau-Aduli, Abdul-Aziz Seidu, Aduli E. O. Malau-Aduli, Theophilus I. Emeto

**Affiliations:** 1 College of Public Health, Medical and Veterinary Sciences, James Cook University, Townsville, QLD 4811 Australia; 2 Department of Population and Health, University of Cape Coast, Cape Coast, Post Office Box UC 182, Ghana; 3 College of Medicine and Dentistry, James Cook University, Townville, QLD 4811 Australia; 4 World Health Organization Collaborating Centre for Vector-Borne and Neglected Tropical Diseases, James Cook University, Townsville, QLD 4811 Australia

**Keywords:** Ashanti region, Ghana, Health care, Integrated health, Traditional medicine

## Abstract

**Background:**

The Ghanaian government has implemented interventions that integrate traditional medicine (TM) into its national health system in response to the high prevalence of TM use. However, empirical evidence of the experiences of service users and the practice of integrated health in Ghana is scanty. Therefore, this study explored the experiences of people with TM integration into the formal health system in Ashanti region using an adapted TM integration framework.

**Methods:**

A sequential explanatory mixed methods study design comprising survey administration and in-depth interviews for data collection was utilised to address the research objective. Framework analysis was used in analysing the qualitative data and for triangulation of results.

**Results:**

Participants were aware of licensing and training of TM practitioners in a science-based university in Ghana. However, knowledge of the existence of TM units in selected hospitals in the region was minimal. Integration knowledge was largely influenced by sex, marital status, household size and residential status, where males and urban dwellers were more familiar with the process than females and rural dwellers. Low patronage of integrated health services in the region was attributable to weak cross referrals. However, service users who had engaged with the integrated system recounted a satisfactory outcome.

**Conclusion:**

Service users’ unfamiliarity with the presence of integrated facilities in Ghana could be an impediment to the practice of integrated healthcare. Sensitisation of the public about the practice of an integrated system could refine the Ghanaian integrated system. Regular evaluation of patient satisfaction and outcome measures might also serve as an effective strategy for improving health services delivery since evaluation is becoming an important component of health service design and implementation. There is the need for future studies to focus on exploring the perceptions and experiences of health practitioners and hospital administrators regarding the practice of integrated health in Ghana.

**Supplementary Information:**

The online version contains supplementary material available at 10.1186/s12913-021-07340-0.

## Background

An integrated health system is a system where healthcare services are expanded through interaction, participation, adaptation and partnership building between orthodox (training, knowledge and method of medicine in westernised cultures [1] and traditional health systems, however indigenous medical knowledge is maintained [2]. In Ghana, TM comprise the use of medicinal plants and faith/spiritual healing [3, 4]. However, in this study, TM was restricted to the use of products such as medicinal plants and plant products used for healing purposes, as this is the only aspect of TM that has been integrated into the formal health system.

Boosting collaboration between the two health systems could help orthodox and traditional medicine (TM) practitioners to complement each other, thereby improving the management/treatment of disease conditions [2]. Integrative medicine has a patient-centred approach to healing and a comprehensive emphasis on healthcare rather than an ailment-centred approach [5, 6]. As oppose to curing, orthodox medicine should aim to adopt a more patient-centred approach to treatment as well. Integrated health system practice has been reported in various countries such as Australia [7], Canada [8], China [9], Israel [6], Ghana [10, 11].

The integration of TM into mainstream health systems is classified into integrative, inclusive, and tolerant systems depending on the degree of the integration, particularly in the areas of health financing, TM regulation, formal education as well as monitoring [12, 13]. Countries such as China Korea, Vietnam, Sri Lanka and Singapore have successfully merged traditional and orthodox health systems [9, 14, 15], and are practising an integrative health system. For example, some Chinese hospitals have TM units where ancient Chinese medicines are used in treating millions of people yearly [14].

A country implements a tolerant health system if the health system is exclusively based on the orthodox health system, nonetheless, some aspects of TM are accepted [12, 13]. Conversely, countries with inclusive health system officially accept TM as a medical practice; however, TM is not entirely integrated into the mainstream health system. For example, formal training on TM at the tertiary educational level might not be available and the traditional health system not included in the country’s health financing scheme [13]. Some developed (Australia, Canada, United Kingdom) and developing (Ghana, Mali, Nigeria) countries practice an inclusive health system [12, 13]. An inclusive health system as practiced in Ghana entails the recognition of TM practitioners as health service practitioners and TM products/medications are accessible at some public health facilities [10, 13]. Hence, traditional and orthodox health practitioners are meant to cooperate and work in a complementarily manner to offer adequate health services to the population [16].

Inclusive health system as practiced in Africa is not efficient due to a number of factors such as weak collaboration between orthodox and TM health practitioners and lack of support from government in terms of training opportunities [4, 17]. This is particularly prevalent in Ghana [4]. Therefore, there is a need to explore factors influencing integrated health systems within Africa with a focus on Ghana.

The government of Ghana initiated the incorporation of TM into mainstream health system through the formulation of a TM policy in 2005, the creation of a TM Practice Council and establishment of TM units in some selected hospitals across the country [10, 18]. Directly related to the creation of TM units, is the fact that recommendations were made by the TM Directorate to public hospitals in Ghana to prescribe TM products to service users [16]. Other interventions introduced include the inauguration of a TM department in a science-based university in Ghana (Kwame Nkrumah University of Science and Technology) in the year 2001 to train TM practitioners [4, 10, 19, 20]. Likewise, an institution established in 1975 was charged with the verification of TM services and product safety before being released into the Ghanaian market, and/or prescribed for human consumption [4, 10, 13].

The government implemented all these interventions to incorporate TM practice into the mainstream health system and ensure its integrity. In 2013, it was reported that approximately 70% of Ghanaians rely on TM for healthcare [21]. The integration of TM into the Ghanaian health system was triggered by the high prevalence of TM use among the populace [22]. In Ghana, TM is used to cure and manage ailments such as fevers, cuts, foot rots, stroke, cancer, and diabetes [10, 21, 23]. In most parts of the country, TM alone is sometimes used in treating malaria, while other times it is used to complement the orthodox anti-malarial therapies [10, 24–26]. For example, nibima a medicinal plant also known as *Cryptolepis sanguinolenta* is a commonly used and scientifically proven treatment for malaria [27]. Nibima’s tea formulations branded as Phyto-Laria, were reported to provide 93.5% healing with no side effects [24, 27]. A Ghanaian study focused on expectant mothers accessing antenatal health services has also recounted the use of TM for the treatment of abdominal discomfort, constipation, safeguarding pregnancies and safe deliveries [28].

Although, TM is widely utilised in Ghana, it is not entirely nontoxic [29]. Careless and unregulated use of TM might jeopardise the health of consumers [30–32]. There are reports of negative health reactions following the use of TM alone or alongside orthodox therapies [33, 34]. For example, research has shown an association between the use of raspberry leaves and increased risk of caesarean delivery [35].

Several African studies have been conducted to examine peoples’ engagement with integrated health system [4, 10, 36]. For example, two previous studies in Ghana assessed the familiarity of service users and health practitioners with TM integration and suggested that engagement with the integrated health system is unsatisfactory [4, 11]. However, these studies had a narrow scope and targeted service users within only one of the piloted integrated facilities in Ghana. Both studies also adopted a qualitative approach in achieving their research objectives and presented mostly descriptive findings that are not supported by a theory. Hence, there is the need to extend the scope of these previous studies by conducting a mixed methods research within communities to grant the majority of Ghanaians the opportunity to present their views on the practice of integrated health system. The Ashanti region is one of the cosmopolitan regions in Ghana with the largest population size of 4, 780, 380 [37]. The region is noted for its diverse socio-economic and cultural backgrounds [4, 37]. Therefore, the current study sought to address the research question: what are the experiences of community members in Ashanti region in relation to TM integration into the health system? The study primarily explored the knowledge, involvement and satisfaction of residents of Ashanti region concerning the practice of integrated health using the conceptual framework for integrating TM into national health systems cited in Park and Canaway [38]. The use of a theoretical framework in this study could enhance the transferability of results and direct the way to more efficient strategies to improve the Ghanaian integrated health system.

### Theoretical framework

The framework for integrating TM into national health systems describes the following four major elements influencing TM integration: Contextual/population characteristics, consumer experience, health governance and financing, and health architecture [38]. The framework for integrating TM into national health systems was developed to explain the role of TM in the Asian Pacific countries and demonstrates how TM integration could lead to the achievement of universal health coverage. The framework was adapted because its constituents were suitable for the study. It recognises population/contextual factors and consumer experiences as important catalysts for successful TM integration. The sections of the framework, which explains integration, was applied to the study.

The contextual/population characteristics describe the influence of demographic features, residential status and notable use of TM on the practice of integrated health. Significant use of TM among a given society could positively influence the integration process. Socio-cultural and economic characteristics vary across countries [38]. Therefore, TM use tend to be extensive in some countries than others. The historic use of TM in a given setting could serve as a catalyst to integration through social influence – peoples’ familiarity with the traditional health system. For example, 40% of all health services delivered in China originate from the traditional health system and the system is used to treat approximately 200 million Chinese every year [12]. Clearly, it is not surprising that China is one of the Asian countries with well-established integrated health system [38].

Consumer experience on the other hand, explores the relationship between service users and the integrative team of orthodox and TM practitioners [38]. This means that an improved integrated system ensures that service users engage with both orthodox and TM practitioners at a formal level rather than independently patronising a blend of the two health systems [38]. Consumer experience is impacted by the accessibility of health systems, knowledge about integration, preference for integration and satisfaction derived from accessing the health systems [38]. To enhance people’s experience in accessing healthcare would require harmony and / or complementary roles of the orthodox and traditional health system since both systems have strengths needed to offer the best of care to service users.

**Fig. 1:**
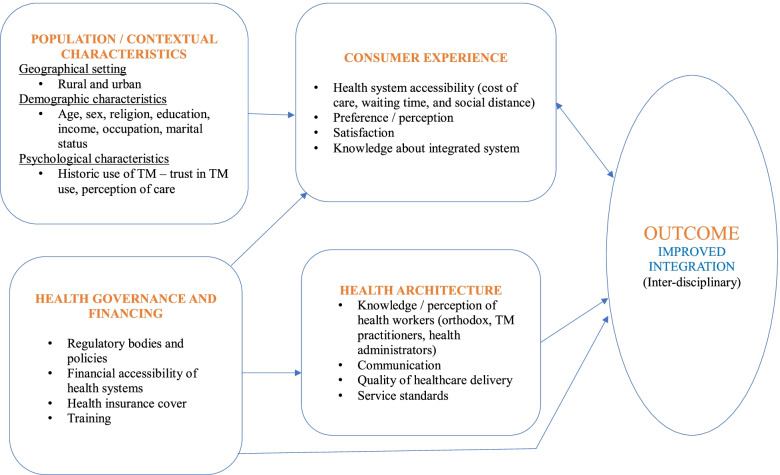
Conceptual framework for integrating TM into national health systems. Source: Adapted from Park and Canaway (2019)

The health governance and financing element focuses on how policy makers influence health systems through funding, education, training and regulations as shown in Fig. 1. For example, governments’ ability to cover TM products and services under national health insurance schemes could boost the integration process by providing continuous funding [38]. Lastly, health architecture talks about the nature of health delivery in a given country. Health practitioners’ appreciation of the role TM plays in health delivery could positively influence the practice of integrated health [38]. That is, the positive attitudes of both TM and orthodox health practitioners towards integration might enhance communication within the system [38]. The current study is part of a larger study assessing the enablers and barriers to TM integration into the Ghanaian health system by exploring the views of community members, health practitioners and hospital administrators in Ashanti region. This paper focuses on only two elements of the framework – population/contextual characteristics and experiences of the community members who are the consumers / users of the integrated health services. The study concentrated on the two components because the influence of TM use and experiences of service users can be assessed adequately through the views of community members / health service consumers, who are the target population for this study. The framework has been used to study TM integration in Asia and the Western Pacific [38]. However, to the best of our knowledge it has not been employed to assess integrated systems in West Africa, specifically Ghana.

## Methods

### Study setting and target population

The Ashanti region is located at the southern part of Ghana and covers 10.2% of the total land areas of Ghana [37]. It shares boundaries with the Brong Ahafo, Western, Eastern and the Central regions. The Ashanti region is divided into 30 administrative districts. Four of the districts are municipalities with one metropolis that is Kumasi, the regional capital [37]. The Kumasi metropolis is surrounded by the Ejisu-Juaben municipal district, Atwima Kwanwoma, Atwima Nwabiagya and Kwabre districts. In the region, the Offinso north district shares boundaries with Offinso south, Mampong and the Ejura-Sekyeredumase districts. The central or strategic location of the region enables the transportation and delivery of goods and services in Ghana and beyond [37]. The majority of the region lies within the semi equatorial forest sector in Ghana.

The Ashanti region has a population of 4, 780, 380 accounting for 19.4% of the total population of Ghana and a population density of 196 residents per square kilometer [37]. Due to the central location of the region, it serves as a destination place for travelers from other parts of Ghana [4]. In view of this, various ethnic groups such as Mole-Dagbon (11.3), Ewe (3.8), Gurma (2.8), Grusi and Mande (2%), Guan (1.5%), and Ga-Dangme (1.2%) have been identified in the region. However, the predominant ethnic group in the region is the Akans representing 74.2% of the total population in the region and the widely spoken language is the Asante Twi [37]. The multi-cultural nature, high population size and diverse socio-economic status in the Ashanti region make findings from researches conducted in the region a reasonable portrayal of what pertains in Ghana.

**Fig. 2:**
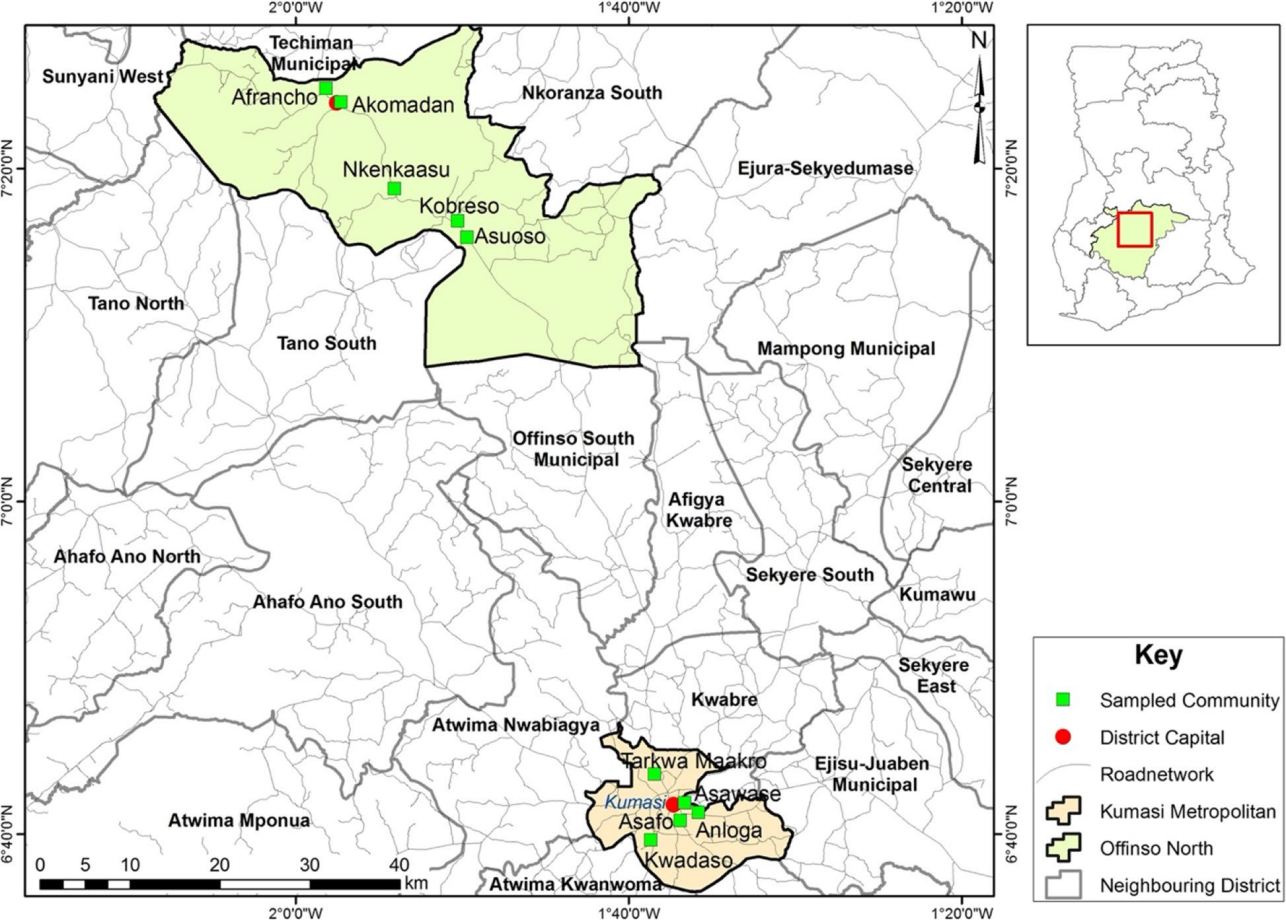
Map of Ashanti region showing the study settings (Kumasi Metropolis and Offinso North district). Source: GIS unit of Department of Geography and Regional Planning [41]

To examine if there are variations in experiences of community members relating to TM integration into the health system, two contrasting districts (Kumasi metropolis and Offinso north district) were selected for the study. Kumasi metropolis was chosen as the urban locality because it is the regional capital and the most populous area accounting for 36.2% (1, 730, 249) of total population of the Ashanti region. Offinso north district was chosen as the rural setting since almost 58.8% of its residents live in agrarian areas [39, 40]. The study population included inhabitants of Kumasi metropolis and Offinso north district who were aged 18 years and above. People who consented to participate were recruited for the study. Overall, 10 communities (5 communities from each district) were randomly selected from the study districts using the electoral registers. For Kumasi metropolis, the communities selected were Anloga, Asawase, Asafo, Kwadaso and Tarkwa Maakro, while Afrancho, Akumadan, Asuoso, Kobreso and Nkenkaasu were chosen from Offinso north (See Fig. 2). The rationale for the selection of these communities was to ensure fair representation of the study settings given that the Kumasi metropolis has been further divided into sub health districts [42, 43].

### Study design

**Fig. 3:**
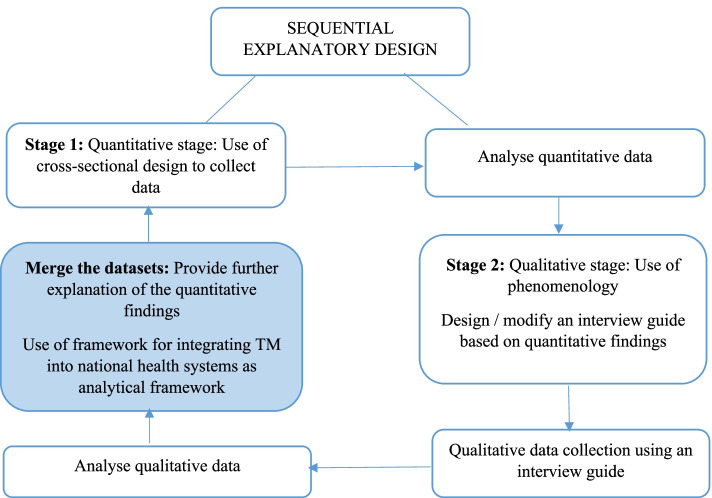
Study design: Sequential explanatory design

A sequential explanatory mixed methods design comprising both quantitative and qualitative methods was employed to achieve the study’s aim. This design applies a systematic merging of quantitative and qualitative research methods within a single study to offer detailed interpretation of results [44]. Explanatory design involves the initial collection and analysis of quantitative data followed by the collection and analysis of qualitative data in order to offer further explanation or expand the first phase quantitative findings [45]. Hence, the first phase of the study focused on the quantitative survey whilst the second phase was a qualitative one. For the quantitative phase, a cross-sectional design investigating demographic and use of TM  was used, while phenomenology (a qualitative design used to describe the lived experiences of people in relation to a specific phenomenon) [46] through individual in-depth interviews was employed in the qualitative component. Thereafter, findings of the quantitative phase informed the development of the interview protocol for the qualitative phase. Qualitative results offered deeper meaning or further explanation of the quantitative findings (See Fig. 3). The mixed methods approach was adopted to strengthen the validity of the study findings by neutralising biases associated with both quantitative and qualitative approaches [44, 47].

### Data collection

Data were gathered from September 2020 to January 2021. Ten research assistants were enlisted and trained by the first author (IGA) to assist in the collection of both quantitative and qualitative data. Research assistants were enlisted from the University of Cape Coast and the Kwame Nkrumah University of Science and Technology. They held bachelors and master’s degrees in the field of Public Health as well as Geography. All the assistants had undertaken some form of research through dissertations and theses writing.

The training was conducted on two separate days and each session lasted for five hours. The study questionnaires and interview guides were used as materials/modules for the training. The assistants were familiar with both quantitative and qualitative research methods and had experience in data collection. However, they were trained to enable them understand the aim of the study and acquaint themselves with the study instruments. The study was executed in two phases, quantitative and qualitative phases.

### Quantitative phase

For the quantitative phase of the study, sample size was calculated using Lwanga, Lemeshow and World Health Organization (1991) formula for sample size determination, given as *n* = z^2^ *pq*/*d*^2^ where p = prevalence of TM use in Ashanti Region, d = level of uncertainty (5%/0.05), z^2^ = 95% level of confidence and *q* = 1−*p* [48]. This formula gave a total of 323 community members as required participants. Therefore, one hundred and sixty-two participants were randomly chosen from the five selected communities within the Kumasi metropolis, while 161 were recruited from the five Offinso north settlements. Systematic random sampling technique (a quantitative sampling technique where the initial unit is randomly selected in an ordered population and subsequent selection is based on a fixed sampling interval from the random start point) [49] was applied in recruiting houses for the study. Participants were then selected from the household units using simple random sampling. In a circumstance where a household had more than one eligible participant, voting was conducted to elect one person.

### Survey instrument

A validated tool, adapted from Allam, Moharam [6] and Adjei [50] was used for quantitative data collection. The adapted instruments have been used in Saudi Arabia and Ghana respectively. The Ghanaian study targeted residents of the Wassa Amenfi district in the Western part of the country. The instrument was a structured questionnaire developed along the lines of the theoretical framework of the current study. It consisted of two major sections – contextual/population characteristics and consumer experience. The population/contextual characteristics section assessed socio-demographic characteristics of participants, while the consumer experience section examined issues related to health systems accessibility, such as attitude towards safe TM practice, preference for integration, knowledge/perception about TM integration and involvement/satisfaction with health systems. Mean scores were computed from patronage, knowledge, attitude, preference and involvement with integrated system survey questions. One point (1) and zero (0) were awarded for the ‘yes’ and ‘no’ responses, respectively. Higher scores indicated positive attitude towards TM integration. For example, participants who reacted positively to all the five preference questions were deemed to have a high inclination towards TM integration [6]. A 2.5 mark was designated as the median score. Therefore, all scores above the median mark were considered high preference for TM integration and the scores below 2.5 inferred low preference. The survey instrument was pilot tested in Suame and Daaso in Kumasi metropolis and Offinso north district, respectively (*n* = 32). Pilot testing was carried out to determine participants’ understanding of the questions before commencement of the actual fieldwork. Participants were surveyed for the quantitative phase of the study. The questionnaires were administered face to face with the help of research assistants. The survey was administered orally. Assistants read and translated the survey questions to participants in the dominant local Twi dialect. Assistants then documented the answers from the participants accordingly. Informed consents were sought from participants before interviews started. Study participants were also assured of confidentiality of the information given. All participants were interviewed in their homes, under conducive environments and the interviews lasted for a period of 30 to 40 min. Results from the quantitative phase were then used in formulating and modifying questions for the qualitative phase. In addition, participants were asked to indicate their interest in participating in the qualitative phase by providing their contact details and availability.

### Qualitative phase

The qualitative phase explored community members’ experiences with the Ghanaian integrated health system. Twenty community members were selected from the 10 communities using purposive sampling procedure. Two research assistants (male, female) who are familiar with qualitative research conducted all the 20 face-to-face individual in-depth interviews. Since the issue under study was not sensitive, gender-based interviews were not conducted. For example, the female participants were not strictly interviewed by the female research assistant. The first author (IGA) trained assistants on the aim of the study using interview guide as training material. A mock interview was then organised between the first author (IGA) and the research assistants to ensure consistency in the interview process. The interview guide was developed by the research team and included topics on health systems accessibility, knowledge about the integrated system and satisfaction derived from accessing the various health systems. The interview guide was pre-tested before actual data collection to ensure that questions were well defined and presented in a logical manner to aid participants’ understanding. Pre-testing was conducted among five people, three from an urban community (Suame) and the remaining from a rural setting (Daaso). Questions were reported to be adequate, understandable with coherent flow of the issues. During the actual data collection, IGA was present in the first four interviews to ensure precision and consistency. There was no communication between IGA and the participants. The research assistants were located within the study area (Kumasi metropolis and Offinso north district) at the time of data collection. Both verbal and written informed consents were sought from all participants before commencement of the interviews. All interviews were conducted face-to-face and participants were interviewed in the comfort of their homes, free from incursions by third parties. The duration of the interviews was between 25 to 45 min and each interview was audio recorded. Data saturation was achieved when explicit concepts/opinions kept iterating [51] after the 16th interview. Nonetheless, assistants interviewed the remaining four participants who had already showed interest in the study to prevent unintended elimination of new concepts. Assistants also made notes on their observations during the data collection period. Repetition of the interview sessions was not needed, but clarifications were sought from some of the participants after the data collection period.

### Data analyses

Quantitative data were analysed using Statistical Package for Social Sciences (SPSS) Version 24 software. Socio-demographic characteristics of participants were presented using descriptive statistics (frequencies and percentages). Associations between categorical variables were determined using Chi square or Fisher Exact test as appropriate. Multivariable regression analysis was ran to identify predictors of preference for TM integration among study participants. All statistical tests were considered significant at a *p*-value of *p* < 0.05.

For qualitative analysis, recorded interviews were transcribed by two qualified transcribers and reviewed by IGA. IGA listened to the interviews and compared them with the transcribed data to ensure preciseness of the data. The transcribed data were read out to the participants during follow-up meetings for authentication and rectification when required. Framework analysis was employed in analysing the transcribed data in NVivo version 12 software. This approach utilises both inductive and deductive analytical techniques and entails five stages; familiarisation, defining thematic framework, indexing, charting, mapping and interpretation [16]. Familiarisation is the stage where the transcripts were read severally, and salient points noted. Thematic framework (identification of main concepts from data) was formed based on notes taken while familiarising with the data. At this stage, key concepts/ideas that had been expressed by the participants were identified through an inductive approach. Indexing, the third stage was carried out by highlighting sections of the data as belonging to certain themes/topics. The authors maintained open minds during the development of thematic framework and indexing stages, which allowed themes to emerge freely. The pieces of marked information were then organised in charts in line with the themes and quotes relevant to the themes were identified. Lastly, mapping and interpretation were performed by organising the charted data to describe participants’ experiences in relation to healthcare accessibility, knowledge/perception about TM integration, and satisfaction derived from accessing the various health systems.

The first eight transcripts were coded and IGA generated initial themes before data collection continued. This was to allow for new probes or questions that incorporated insights gained after an initial review of the data. To increase credibility and trustworthiness of findings, transcribed data, codes and themes were independently assessed by BSMA. Additionally, data were crosschecked, and a 90% degree of consistency existed between both authors’ identification of themes, coding and classification. Discrepancies were settled through discussion and mutual agreement. The other three authors (AEOMA, AAS and TIE) reviewed the quotes and themes to enhance trustworthiness of the results. Themes were presented together with illustrative quotes attached with participants’ socio-demographic characteristics (for example, Participant 1, Afrancho, female, 49 years). The performance of an inter-coder reliability enhanced the rigour and transparency of the study analysis and its application to the data [52]. The COREQ checklist [53] for reporting qualitative studies was used to appraise the final version of the qualitative aspect of the manuscript (See Supplementary file 1, COREQ Checklist).

### Positionality statement

According to Scharp and Thomas [54], critical researchers should evaluate their own experiences, which might contribute to the interpretations of the experiences of other people. In light of this, none of the authors have experienced or patronised integrated health services in the study area. Instead, the first author has conducted a research on the utilisation of healthcare services among residents of a Ghanaian community and reported that TM use was prevalent and appeared to be the preferred healthcare system among the study population. The first author maintains that having this knowledge about preference for TM makes her relate to the study participants’ desire for TM integration. More so, not identifying as TM users or advocate for TM use permitted the research team to protect themselves from being bias in the interviewing process as well as the presentation of study participants’ experiences. To avoid speaking for the data, notes were made on all presumptions that arose about the target population and study setting. This was done to bracket the existing suppositions during the data collection exercise and data analysis procedure.

### Ethics

The Ghana Health Service Ethics Review Committee (GHS-ERC003/05/20) and the Human Ethics Committee at the James Cook University, Australia (H8239) granted approval for this study. During the data collection, both written and oral informed consent were obtained from all the participants. In addition to the ethical approval and informed consent, all methods were performed in accordance to the Declaration of Helsinki on ethical principles in conducting human research.

## Results

### Quantitative phase

#### Contextual/population characteristics

##### Socio-demographic of participants

A total of 323 participants completed the survey. The socio-demographic attributes of the participants are presented in Table 1. The mean age was 33.6 ± 15.9, with a range of 18 to 80 years. The majority of the participants were within the ages of 20-29 years (42.7%). Males constituted 52.6% of the total population. Most of the participants (58.2%) were not married. Only 9% attained tertiary level of education, 34.7% completed secondary/senior high school education, primary education (9%) while 18% had no formal education. A greater proportion of the participants were traders (30.7%), while only 4% were government employees. Christianity is the dominant religion in the study area (74.3%). Participants were mostly (65.3%) from the Akans ethnic group. Half (50.2%) of the participants reside in urban settlements. A majority of households (59.1%) comprise of five or more members. Most, of the study participants (79.2%) had a household monthly income of less than 1000 GH Cedis (USD 172.4) (Table 1).

**Table 1:** Socio-demographic characteristics of study participants (*N* = 323)

Variables	Frequency (n)	Percentage (%)
**Sex**		
Males	170	52.6
Females	153	47.4
**Age (years)**		
Range: 18 – 80		
Mean ± SD: 33.6, 15.9		
Below 20	44	13.6
20-29	138	42.7
30-39	54	16.7
40-49	30	9.3
50+	57	17.7
**Marital status**		
Unmarried	188	58.2
Ever married	135	41.8
**Educational level**		
Secondary/Senior High School	112	34.7
Middle/Junior High School	95	29.4
No formal education	58	18.0
Primary	29	9.0
Tertiary	29	9.0
**Occupation**		
Trading	99	30.7
Other Specify (driver, mechanic, shop attendant)	68	21.1
Artisan	62	19.2
Student	51	15.8
Farming	29	9.0
Government employee	14	4.3
**Religion**		
Christian	240	74.3
Non-Christian (Islam and Traditional)	83	25.7
**Ethnicity**		
Akan	211	65.3
Mole Dagbani	98	30.3
Ga/Ewe/Guan	14	4.3
**Geographical location**		
Urban (Kumasi metropolis)	162	50.1
Rural (Offinso North)	161	49.9
**Household size**		
5 or more	191	59.1
Below 5	132	40.9
**Household monthly income (GH Cedis)**		
0-499	148	45.8
500-999	108	33.4
1000-1499	40	12.4
1500+	27	8.4

*GH* Ghanaian, *SD* standard deviation

### Health system accessibility

As shown in Table 2, the majority (98.5%) of participants perceived traditional medicine to be culturally acceptable. Likewise, 54.5 and 56.7% reported that TM was more accessible from a geographical location and monetarily point of view, respectively.

**Table 2:** Healthcare accessibility among participants

	Frequency (n)	Percentage (%)
**Financially accessible medical system**		
TM system	183	56.7
Orthodox system	140	43.3
Total	323	100
**Geographically accessible medical system**		
TM system	176	54.5
Orthodox system	147	45.5
Total	323	100
**Culturally acceptable medical system**		
TM system	318	98.5
Orthodox system	5	1.5
Total	323	100

### Patronage, knowledge, attitude, preference about traditional medicine integration, and engagement with the integrated health system

The quantitative findings on participants’ patronage of, knowledge about, preference for, attitude to TM and engagement with the integrated health system are shown in Table 3. All constructs had acceptable reliability or internal validity as determined by Cronbach’s alpha; attitude (0.673), preference (0.820), knowledge (0.6885), experience (0.699) and patronage (0.385).

**Table 3:** Participants’ patronage, knowledge, preference, experience and attitudes towards traditional medicine practice and its integration into formal the health system

Variables/Questions	Yes		No	
	n	%	n	%
**Patronage of TM**				
Have you ever used TM? (By TM, I mean the use of plant seeds, berries, roots, leaves, bark, flowers for medicinal purposes)	307	95.0	16	5.0
Do you seek health advice from TM practitioners?	183	59.6	124	40.4
Do you ask your physician about TM when you want to use them?	15	5.0	292	95.0
Do you ask the pharmacist about TM when you want to use them?	12	3.9	295	96.1
**Knowledge about TM integration**				
Do you have knowledge about the incorporation of TM into health system?	214	66.2	109	33.8
Is there a license for TM practice in Ghana health system?	205	95.8	9	4.2
Are there laws to regulate TM in Ghana?	205	95.8	9	4.2
Are you aware of the introduction of TM directorate in some hospitals in Ghana/Ashanti region?	99	46.3	115	53.7
**Attitude towards safety of TM practice**				
Should TM container have a warning of possible side effects and interaction with other medications?	323	100.0	0	0.0
Should TM container have a clear note if the medicine is approved by FDA as a safe medication?	322	99.7	1	0.3
Should TM container be labelled with the name of active ingredients, required dose and instruction on when to use?	321	99.4	2	0.6
Should TM container be labelled with the expiry date?	321	99.4	2	0.6
Should TM container have a license and registration number?	320	99.1	3	0.9
Does the production and selling of TM products need to be regulated by Ministry of Health?	317	98.1	6	1.9
Should TM practitioner be certified from the Ministry of Health?	316	97.8	7	2.2
Should TM practitioner have a degree in this profession?	270	83.6	53	16.4
Do you think the pharmacist can give useful advice to you if you want to use TM?	209	64.7	114	35.3
**Preference for TM integration**				
Do you prefer TM integration into the formal health system?	314	97.2	9	2.8
Do you want your physician to follow up when you are using TM to avoid any side effect?	312	96.6	11	3.4
Do you want your physician to give you advice about safe use of TM?	260	80.5	63	19.5
Do you think a physician can monitor your health better if he/she knows the kind of TM you are using and who prescribed it?	256	79.3	67	20.7
Would integrating TM practice into health system make you feel safer to use TM?	249	77.1	74	22.9
**Engagement with the integrated health system**				
Have you ever been referred by a medical doctor to a TM practitioner?	23	7.1	300	92.9
Have TM ever been prescribed for you at the hospital/clinic by a medical doctor?	10	3.1	313	96.9
Have you ever been referred by a TM practitioner to a medical doctor/hospital/clinic?	7	2.2	316	97.8
Have orthodox medicines ever been prescribed for you by a TM practitioner?	4	1.24	319	98.8

### Patronage of and knowledge about traditional medicine integration

An overwhelming majority (95%) of the participants had used TM. However, nearly none of them consulted their physicians (95%) or pharmacists (96.1%) before TM usage. Additionally, only 59.6% of the participants who had used TM reported that they sought healthcare advice from TM practitioners, meaning some participants used TM based on their own discretion. Participants had some form of knowledge about TM integration into the Ghanaian health system. Most (95.8%) participants were aware of licensing and existence of laws governing TM practice in Ghana. Nonetheless, only 46.3% had knowledge about the presence of TM directorates in selected hospitals in Ashanti region and Ghana as a whole.

### Attitude towards safe traditional medicine practice

The majority of participants believed that TM practitioners should have a degree in the profession (83.6%) and should acquire certification from the Ministry of Health before they are allowed to practice (97.8%). Ninety-eight percent (98%) of participants insisted that the production and sale of TM products needed to be controlled by the Ministry of Health. Almost all the participants (99.1%) felt that TM products should have a license and registration number. All participants, (99.4%) believed that TM containers/packages must specify names of active ingredients, required dosage, expiry date and instructions on how and when to use the product. They (99.7%) also believed that TM products should be labelled with an approval note from the Food and Drug Authority (FDA). All study participants (100%) maintained that TM containers/products should have a warning of possible adverse effects and contraindications. A majority of participants (64.7%) felt pharmacists could offer useful advice about traditional therapies.

### Preference for traditional medicine integration

Most participants demonstrated a positive preference for TM integration. For example, 96.6% want physicians to follow up/check with them when they are using TM to avert side effects; 80.5% want to receive advice from physicians about safe usage of TM products/services and 79.3% felt a physician can monitor their health better if the physician knows the type of TM they use and the prescriber. A significant percentage of participants (77.1%) would feel safer to patronise TM products and services if traditional therapy was properly integrated into the formal health system.

### Engagement with the integrated health system

Generally, it was reported that cross-referrals rarely occurred between orthodox and TM practitioners in the Ashanti region. When participants were asked about their interaction with the integrated system, 92.9 and 97.8% of participants had never been referred by a medical doctor to TM practitioners or vice versa, respectively. In terms of prescription of medication, 96.9% of participants recounted that TM had never been prescribed to them at hospitals/clinics or by a medical doctor. Likewise, 98.8% also stated that orthodox medicine had never been prescribed to them by a TM practitioner.

### Satisfaction from health systems

Of the total sample, 81.4% of participants indicated that traditional therapies are highly effective, while 9% were neutral (Table 4).

**Table 4:** Satisfaction from health systems based on effectiveness

Effectiveness of health systems		
	Frequency (n)	Percentage (%)
TM system	263	81.4
Orthodox system	31	9.6
Indifferent	29	9.0
Total	323	100

### Socio-demographic characteristics and knowledge about TM integration

Table 5 shows the association between socio-demographic characteristics of participants and knowledge about TM integration into the Ghanaian health system. There was sufficient evidence of a positive association between knowledge about TM integration and sex (*p* < 0.001), marital status (*p* = 0.013**)**, geographical location (*p* = 0.001), household size (*p* < 0.001.). This implies that more males (76.5%) than females (54.9%) knew of the integration process; unmarried participants (71.8%) exhibited more knowledge about TM integration than their married counterparts (58.5%). Similarly, majority of the urban dwellers (75.3%) and those with less than five household members (78.8%) were more knowledgeable about TM integration than the rural residents (57.1%) and participants with five or more household members (57.6%).

**Table 5:** Socio-demographic characteristics and knowledge about TM integration into health system

Variables	Yes		No		***p***-value
	n	%	n	%	
**Sex**					**< 0.001**
Male	130	76.5	40	23.5	
Female	84	54.9	69	45.1	
**Age**					**0.731**
Below 20	30	68.2	14	31.8	
20-29	96	69.9	42	30.4	
30-39	35	64.8	19	35.2	
40-49	19	63.3	11	36.7	
50+	34	59.6	23	40.4	
**Marital status**					**0.013**
Unmarried	135	71.8	53	28.2	
Ever married	79	58.5	56	41.5	
**Educational level**					**0.378**
Secondary/Senior High School	78	69.6	34	30.4	
Middle/Junior High School (JHS)	66	69.5	29	30.5	
No formal education	34	58.6	24	41.4	
Tertiary	20	69.0	9	31.0	
Primary	16	55.2	13	44.8	
**Occupation**^**a**^					**0.901**
Trading	63	63.6	36	36.4	
Other Specify	47	69.1	21	30.9	
Artisan	42	67.7	20	32.3	
Student	35	68.6	16	31.4	
Farming	17	58.6	12	41.4	
Government employee	10	71.4	4	28.6	
**Geographical location**					**0.001**
Urban (Kumasi metropolis)	122	75.3	40	24.7	
Rural (Offinso north)	92	57.1	69	42.9	
**Religion**^**a**^					**0.055**
Christianity	167	69.9	73	30.4	
Islam	46	57.5	34	42.5	
Traditional	1	33.3	2	66.7	
**Ethnicity**					**0.126**
Akan	148	70.1	63	29.9	
Mole Dagbani	58	59.2	40	40.8	
Ga/Ewe/Guan	8	57.1	6	42.9	
**Household size**					**< 0.001**
5 or more	110	57.6	81	42.4	
Below 5	104	78.8	28	21.2	
**HH monthly Income (GH Cedis)**^**a**^					**0.119**
0-499	99	66.9	49	33.1	
500-999	66	61.1	42	38.9	
1000-1499	26	65.0	14	35.0	
1500+	23	85.2	4	14.8	

^a^Fisher exact test

### Predictors of preference for traditional medicine integration into formal health system

A multivariable regression analysis was used to determine predictors of preference for TM integration into the health system adjusting for all possible confounders (Table 6). Household sizes of five or more and lower incomes of 500-999 were associated with preference for TM. Thus, in comparison to household sizes less than five, the odds of household sizes of five or more having a high preference for TM integration was 0.47, 95%CI (0.23, 0.95), *p* = 0.034. Similarly compared to other income brackets, odds of participants who earn monthly incomes between 500 and 999 having high preference for TM integrations was 0.37, 95%CI (0.18, 0.75), *p* = 0.006. Male participants and unmarried participants were more likely to have a high preference for TM integration, but there was insufficient evidence to reject the null hypothesis.

**Table 6:** Predictors of preference for traditional medicine integration into health system

Multivariable analysis			
Variable	AOR	[95%CI]	*p*-value
**Sex**			
Male	1.81	[0.96-3.41]	0.067
**Age**			
Age	0.98	[0.96-1.00]	0.222
**Marital status**			
Unmarried	2.06	[0.85-5.02]	1.112
**Household size**			
5+	0.47	[0.23-0.95]	0.034
**Income (GHC)**			
500-999	0.37	[0.18-0.75]	0.006
1000-1499	0.44	[0.17-1.18]	0.104
1500+	0.67	[0.20-2.31]	0.528

*AOR* Adjusted Odds Ratio *CI* Confidence Interval *GHC* Ghana Cedis

### Qualitative phase

Twenty participants comprising 11 males and 9 females, aged between 20 and 81 years were involved in the interviews. Eight themes emerged from the data. These themes have been mapped under two constructs of the framework – psychosocial factors (trust in TM use, modernised TM products/services, quality of care) and consumer experience (healthcare accessibility, preference/perceived benefits of integration, knowledge about TM integration, satisfaction derived from health systems, recommendations for better integration).

### Psychosocial factors

#### Trust in TM use

Participants irrespective of their geographical location stated that they trusted the use of TM and attributed their trust to the ‘natural’ nature of TM and its ability to offer total healing for one’s health condition without side effects.

*“I trust TM a lot because they are natural, it does not give side effects and it cures our sickness completely.”*

[Participant 3, Akumadan, Female, 45 years]

*“The TM are the best and they are natural. I trust the usage. They help in total healing from a disease a person might be suffering from without any side effects.”*

[Participant 15, Kwadaso, Male, 64 years]

#### Modernised TM products/services

Furthermore, participants described services offered by the traditional health system to be improved. They indicated that current TM practice in Ghana is modernised with advanced ways of processing and packaging TM products as well as the use of machines in diagnosing diseases before treatment.

*“TM these days are not like what it used to be in the olden days. We are now in modern Ghana and everything has been modernised. Things have changed. When you look at some TM, the way they have packaged them tells you that things have really changed. It is not like the time that they only put it in a pot for you and you have to just boil it and be taking it every day. Now, they make some like capsules and when you go to the traditional herbal clinics, they have machines that they use to check you before they even give you drugs. Pre Nkwa herbal centre for instance has a lot of machines there. They treat you just like you have been to an orthodox hospital.”*

[Participant 14, Kobreso, Male, 38 years]

*“Almost all the TM practitioners are also using the modern method of processing drugs. Now, they have a lot of machines to detect diseases.”*

[Participant 8, Anloga, Male, 81 years]

#### Quality of service

Majority of the participants recounted that TM practitioners have a more humane attitude towards patients than their orthodox counterparts. Thus, they (patients) receive quality healthcare when they patronise services at TM centres. To users, this humane attitude of TM practitioners serves as a catalyst for therapy usage.

*“When you go to the TM centres, they will keep calm and listen to you and even ask you to be using the TM the way they have asked you to do. They will pamper you and you will feel very happy. That one alone motivates you to use the medicine they have given you unlike the hospitals where from the nurses to the pharmacies all of them will be shouting at you like you are not a human being.”*

[Participant 2, Afrancho, Female, 50 years]

*“The orthodox health providers don’t care about human beings. When you go and you are dying, they will allow you to die. The nurses especially are not respectful…I told you that I have been visiting a TM centre right; the people there are very good. From the nurses to the doctor and even the security man there. They are all very good and treat clients with much respect.”*

[Participant 20, Tarkwa Maakro, Male, 65 years]

### Consumer experience

#### Health system accessibility

Three issues relating to healthcare accessibility were identified. These issues include availability of services, financial accessibility and faster delivery of service. Participants’ choice of healthcare was largely influenced by these factors. We found that the majority of the participants regardless of their geographical location deemed traditional health system more accessible in terms of physical location.

*“Now, we have TM clinics and they are all close. They sell TM products in cars and in every corner.”*

[Participant 6, Anloga, Female, 40 years]

*“Hospitals can recommend certain medicines for you and you will roam from pharmacy to pharmacy without getting the medicine unless you pick a car to places like Kumasi or Accra. It makes it difficult hence inaccessible. However, a TM doctor can just go to the bush, gather some plants, and prepare for me. Therefore, the TM is more accessible to me.”*

[Participant 4 Akumadan, Male, 24 years]

When discussing the financial accessibility of health systems, participants stressed that, orthodox health services tend to be more economical due to the presence of the National Health Insurance Scheme.

*“The hospital that is the orthodox system since they are covered by the government (health insurance), I do not have to spend much money. The government have taken care of some of the cost so when I add something small (my money) I get the drugs I need.”*

[Participant 13, Asuoso, Male, 43 years]

In addition, some participants clarified that the cost of TM is dependent on the nature of practice. Thus, TM tends to be cost-effective when delivered within informal settings (community-based practice). However, services are expensive when offered in formalised settings such as clinic because TM products and services are uninsured.

*“You see there are TM centres that have been opened like a hospital or clinic…When you go to such places, they take a lot of money from you because of what they do. Their clinics are privately owned and they do not get money from the government (not under health insurance scheme), so they do everything by themselves and that makes their services more expensive but TM that are offered by those in houses are really cheap.”*

[Participant 12, Asuoso, female 38 years]

One of the biggest incentives to accessing traditional health system as stated by participants is the faster delivery of service/care. This makes traditional healthcare desirable to the populace.

*“If it is about how quickly you get access to care, then the TM centres are the best. When you get there, they attend to you on time. All the test they have to do they make sure they do it fast for you. The TM clinic that I visited, there was a queue but it is not as long as the one at the hospitals. As for the hospital, when you are going make up your mind that you are going to spend the whole day there. There is always a long queue there and you do not get treatment on time.”*

[Participant 20, Tarkwa Maakro, Male, 65 years]

*“When I go to the TM clinic, I don’t waste time. They attend to me quickly whenever I go there. The queue is not long so you get the chance to meet the doctor on time. This makes accessing care at TM centres very pleasant to me”*

[Participant 10, Asawase, Female, 80 years]

#### Preference/perceived benefits of TM integration

Participants expressed their preference for TM integration and perceived that integrating TM into the Ghanaian health system could lead to generation of income and preservation of indigenous medicine. While participants from urban setting focused on the financial benefits, participants from the rural setting emphasised the preservation of indigenous medicine.

*“It will help us as a country. It will help to reduce the amount of money we spend on drugs that are imported into the country. If we are using our own TM, then there would not be the need to import a lot of the foreign drug into the country. TM practitioners will also get money because they will be employed to work in the various hospitals. Therefore, both their products and services will be marketed and they will earn money.”*

[Participant 16, Kwadaso, Male, 43 years]

*“Through integration, the TM would not fade out of the system. It will help us to preserve our indigenous medical knowledge.”*

[Participant 14, Kobreso, Male, 38 years]

#### Knowledge about TM integration

The study showed that participants had limited knowledge about TM integration into the Ghanaian health system. Participants were familiar with the recommendation of TM to patients by medical doctors, training of TM practitioners at the Kwame Nkrumah University of Science and Technology (KNUST) and licensing of TM practitioners. The media, particularly television and radio were reported to be the common source of information.

*“Ok it has been going on because when you go to the hospital and you have a disease like Hepatitis B, they (medical doctors) can recommend TM for you”*

[Participant 4, Akumadan, Male, 24 years]

*“I am aware some people are being trained at KNUST to become TM doctors. I got to know about it from a doctor who was on radio. He said it when he was talking about TM practice in Ghana, so for that one I know.”*

[Participant 17, Kwadaso, Male, 50 years]

*“Oh! I have heard of registration of TM practice on radio and television. I know some of the TM practitioners in Ghana here are working with licenses. The TM practitioners who are working underground are those who do not have licenses.”*

[Participant 11, Asawase, Male, 24 years]

Nonetheless, the majority of participants were unfamiliar with the presence of integrated health facilities in Ghana. Despite participants’ exposure to the media, many of them had no clue about the presence of TM clinics in some selected public hospitals in the Ashanti region and the country as a whole.

*“I have no news about TM clinics situated at hospitals. I always listen to the radio and television but I have not heard anything like that before.”*

[Participant 7, Anloga, Female, 44 years]

*“I do not know health facilities that have some (TM units). I am not even sure that they have done the integration.”*

[Participant 18, Nkenkaasu, Female, 24 years]

#### Satisfaction derived from health systems

Participants measured their satisfaction based on efficient collaboration between health practitioners in administering care and effectiveness of therapy. Two themes emerged: helpful collaboration between health practitioners and potency of TM. Although, most of the participants support the practice of integrated health, only few have had the opportunity to interact with the system. The few participants who have, utilised integrated healthcare recounted positive and satisfying outcomes. Thus, users of the integrative system demonstrated that the merging of the two health systems could offer timely and appropriate healthcare to the populace.

*“I was happy the medical doctor referred me to a TM provider. The reception alone from the two providers was just great. The initiative of the medical doctor to refer me to the TM doctor and the timely and appropriate treatment from the TM provider were extremely fulfilling to me. You can see that through the proper interaction between the two providers, I am now very healthy again.”*

[Participant 3, Akumadan, Female, 45 years]

*“The cross referral has helped me a lot. I do not know what would have happened to me if the specialist at Okomfo Anokye did not refer me to the TM doctor. In fact, the way both practitioners came together and offered the best of care was excellent. The specialist did not look down on the abilities of the TM doctor and indeed the TM provider also lived up to expectation. Now, I am healthy again due to communication between these two health providers.”*

[Participant 20, Tarkwa Maakro, Male, 65 years]

Other participants also gave a positive account regarding the use of the traditional health system. According to them, TM are potent in treating maladies.

*“When I got pregnant to my third child, I was having pressure (hypertension). It was very serious and I did not know what to do. I was always sending it to the hospital and they always gave me drugs that will help me but I was not really seeing any serious changes. I was still in pain until I took a TM…When I took it, I could see a lot of changes in my body. The pressure has stopped.”*

[Participant 6, Anloga, Female, 40 years]

*“I felt sick and I was asked by a friend to see a TM provider so I went to the TM centre. The TM was good….I took it and within the first week, I started seeing improvement. The TM was really good for me and I was healed from that health problem totally.”*

[Participant 18, Nkenkaasu, Female, 24 years]

#### Recommendations from participants to promote proper integration of the two health systems

**Fig. 4:**
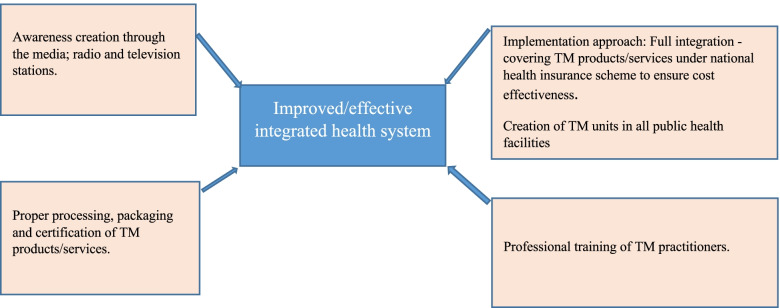
Participants' recommendations to ensure proper integration of TM into the health system

To wrap up, participants shared their opinions on how the integrated system could be improved. These recommendations include sensitisation of the public about the operation of an integrated system through the media, proper processing, packaging and certification of TM products/services, style of implementation and professional training of TM practitioners. Figure 4 presents a summary of the recommendations suggested by study participants.

*“When you implement an intervention and you advertise it, it makes people aware of the existence of that intervention. When people do not know, they wouldn’t use it so they (policy makers) should make sure the advertisement becomes more through the media particularly radio and television stations.”*

[Participant 11, Asawase, Male, 24 years]

*“When you look at containers of TM products from countries like China, you will notice that they have written the expiring dates of the drugs on containers. Some of the TM here do not have that. Some of them are just packaged in plastic bags…they do not look attractive at all and they do not have any description on them too. No expiring date, nothing! Practitioners just write the names of the drugs, and what they are meant for on pieces of papers and that is it. They will not write the expiring date and even how to take them (dosage). It makes using them a bit dangerous. Therefore, they should package them very well. They should write the expiring dates and even how the drugs should be used. That way, it will meet the standard and can easily fit into the formal health system.”*

[Participant 3, Akumadan, Female, 45 years]

*“They have to train more people in the field of TM and make sure that they are good at what they do. Then, post them to the various hospitals and clinics. Now, TM doctors are not many but if they train more people, there will be more experts in the field and every health facility will have a TM doctor. Therefore, getting access to an integrated health facility/care will be easy.”*

[Participant 10, Asawase, Female, 80 years]

Currently, Ghana is practicing an inclusive integrated health system. When participants were asked to suggest an appropriate integration approach, most of them opted for full integration/inclusion of TM in the national health insurance scheme. They envisaged that a fully integrated health system has the advantage of making healthcare geographically and financially accessible to all.

*“Integration should be done as a whole. It should be a nationwide thing….They should not say that let us integrate it at bigger facilities such as Okomfo Anokye teaching hospital and leave the smaller hospitals behind. They should take it to every hospital in the country. If this is done, everyone regardless of place of residence can get access to proper healthcare.”*

[Participant 5, Akumadan, Male, 57 years]

*“TM offered at clinics are very expensive. If you do not have money, you cannot really patronise their services. Therefore, they should make it in such a way that TM products/services will be covered under the national health insurance scheme. That way, insurance will cover some of their charges just like it covers that of the hospitals/orthodox healthcare. If we do that, it will help everybody.”*

[Participant 7, Anloga, Female, 44 years]

#### Triangulation of study results

Table 7 presents the integration of quantitative and qualitative findings informed by the conceptual framework for integrating TM into national health systems.

**Table 7:** Merging of survey and qualitative results, guided by framework for TM integration

Domain of the framework for TM integration into national health systems	Concept/theme	Concept/theme description	Quantitative findings	Illustrative qualitative response:
**Contextual characteristics** **(psychosocial factors):** Contextual characteristics/psychosocial factors describe the historic use or trust associated with TM usage in a given society.	Trust in TM use	Significant use of TM among residents of Ashanti region. Key reason cited for high use of TM among participants was trust in TM due to its natural state and negligible side effects.	High usage of TM among participants: Yes = 95.0% No = 5.0%	*I have much confidence in traditional therapies because you do not get any problems after taken them and it heals you completely, so I use them a lot.* [Participant 18, Nkenkaaso, Female, 24 years]
**Consumer experience:** Consumer experience is influenced by health system accessibility – physical, financial, cultural [38]	Physical availability of healthcare	Participants narrated how healthcare is geographically available to them.	The majority of participants considered TM geographically accessible. TM = 54.5% Orthodox = 45.5%	*The TM are very close. They take it around and if you want it, you just buy and use it. As for the orthodox medicine, no one will bring it to your shop or your work place. You have to look for pharmacy shop and buy.* [Participant 1, Afrancho, Female, 49 years]
	Culturally acceptable healthcare	Furthermore, TM appeared to be the traditionally acceptable health system among participants.	A considerable percentage of participants deemed TM as a culturally acceptable medical system: TM = 98.5% Orthodox = 1.5%	*In the olden days…there were no hospitals and no clinics. Everything concerning our health was dependent on TM. When you are sick, they tell you that take traditional drugs and we are still using it. So, TM to me is culturally acceptable.* [Participant 20, Tarkwa Maakro, Male, 65 years] *Now, we have TM*
	Financial accessibility	Cost of care was dependent on nature of services delivered. In that, modernised TM practice was reported to expensive, while local TM services were deemed economical.	More than half of participants recounted TM to be less expensive: TM = 56.7% Orthodox = 43.3%	*clinics and they have made it like the hospitals with their nurses and others…those TM clinics are expensive but if you visit an old woman in the house to prepare some TM for you, that one is less expensive.* [Participant 19, Nkenkaaso, Male, 20 years]
**Consumer experience:** Consumer experience is impacted by satisfaction derived from utilising the various health systems as well as motivation for usage [38].	Satisfaction from health systems	Satisfaction from health systems was based on effectiveness of therapy.	More than three-quarters of the participants reported that they gain satisfaction from accessing TM because it is effective in treating ailments. TM = 81.4% Orthodox = 9.6% Indifferent = 9.0%	*The best medicine I can talk about is TM. If not for TM, I know I would not be alive by now. I was very sick. It was not easy for me at all but TM has saved my life.* [Participant 10, Asawase, Female, 80 years]
**Consumer experience:** Consumer experience is influenced by knowledge about the integration process [38].	Knowledge about TM integration	Participants demonstrated their familiarity with the integration process. Knowledge about integration varied among sex of participants.	More males (76.5%) than females (54.9%) were aware of TM integration into the Ghanaian health system *p*-value < 0.001	*So yes, I have heard that TM has been integrated into our healthcare system. I am a man so I keep track of issues especially health related issues. Even our current president Nana Addo met some of the experts to find out from them how well they can implement that. So, I am aware of it.* [Participant 17, Kwadaso, Male, 50 years]
		Knowledge about integration differed in terms of marital status of participants.	Participants who were not married (71.8%) exhibited more knowledge about TM integration than their ever married counterparts (58.5%) *p*-value = 0.013	*I know there are some pharmacies that sell TM products. When you visit such a facility, the TM provider will tell you to go to the hospital for the doctors to examine you before he starts treatment. That way, the provider will be sure of what you are suffering from and know the kind of drugs to give to you. I am young and single oo but I know a lot of things about health* [Participant 19, Nkenkaasu, Male, 20 years]
		Participants’ residential status influenced their knowledge about TM integration.	A greater proportion of urban dwellers (75.3%) were more knowledgeable about TM integration than the rural residents (57.1%). Hence, being a city dweller was perceived to be advantageous. *p*-value = 0.001	*Oh yes, I have heard about TM integration on the radio, that is Peace FM. My brother, I feel lucky to be in the city because any new intervention starts from the city…They said that, now the hospitals have been made in such a way that when you visit the facility and you prefer TM, they will send you to a TM centre to be treated there. For instance, if you are suffering from malaria, they have some TM at the hospital that can treat malaria and they will prescribe that for you.* [Participant 16, Kwadaso, Male, 43 years]
		The size of participants’ households influenced their knowledge about integration.	Participants with less than five household members (78.8%) were familiar with TM integration than those with five or more household members (57.6%). *p*-value = < 0.001	*Currently, there are people at KNUST who are learning TM. That is what they have gone to school to study. We are only two in this house, I told you one of my grandchildren is staying with me and he is the one who told me. He said it when I was sick and receiving care at a TM centre. Therefore, many facilities will have it (TM units) in few years to come.* [Participant 20, Tarkwa Maakro, Male, 65 years]
**Consumer experience:** Consumer experience is shaped by people’s preference for integration [38].	Preference for TM integration	Larger household as a predictor of preference for TM integration. Participants with larger households were more likely to choose TM integration.	In comparison to household size less than 5, the likelihood of service users with household size five and above having preference for integration is [0.47; 0.23-0.95] *p*-value = 0.034	*I support integration with all my heart because with integration people like me who have larger families can have access to good healthcare. I have a large family my sister! In all, we are nine that is wife, seven children and myself. So do you understand why I prefer integration?* [Participant 13, Asuosu, Male, 43 years]
		Participants who had lower household monthly income have a high propensity to prefer integration.	The possibility that a participant who earned between 500 and 999 Ghana Cedis to prefer TM integration was lower than those who earned below GHC 500. 500-999: [0.37; 0.18-0.75] *p*-value = 0.006 1000-1499: [0.44; 0.17-1.18] *p*-value = 0.104 1500+: [0.67; 0.20-2.31] *p*-value = 0.528	*I prefer integration because if I go to the hospital and they are unable to cure me, then I can get treatment from a qualified TM doctor without having to spend much. I do not earn much; I earn just 300Gh Cedis a month so through TM integration, even with my little income, I will get proper care and can patronise quality TM products and services when the need arises.* [Participant 14, Kobreso, Male, 38 years]

## Discussion

This study investigated community members’ experiences with TM integration into the Ghanaian health system using Kumasi metropolis and Offinso north district as study sites. The contextual/population characteristics and consumer experience components of the framework for integrating TM into national health systems [38] provided the theoretical underpinning for this research. Majority of the study participants were familiar with TM use and attributed their fondness for traditional therapies to its ‘natural’ nature and minimal side effects. They also noted the advanced nature of TM practice in Ghana. The high prevalence of TM use as reported in this study is consistent with the findings of Yarney, Donkor [21], which stated that 70% of people residing in Ghana patronise TM. Earlier studies in Ghana also reported high prevalence of TM use among expectant mothers, cancer patients, HIV positive patients and urban-periphery settlers [21, 28, 43, 55]. The immense use of TM and improved nature of the practice in the Ashanti region and Ghana as a whole could be an incentive to the integration process because significant and cultural use of TM is identified as one of the indicators/contextual factors needed to promote successful TM integration into mainstream health systems [38].

The study established that access to healthcare is dependent on physical, financial and cultural factors. The common presumption in the integration discourse is that people in middle/low income countries continue to utilise TM because such medicines/services are inexpensive [12], but this is arguable in our findings. The study suggests that the cost of TM is dependent on the nature of practice. Thus, modernised TM products/services are deemed costly owing to absence of insurance cover. However, community-based TM practice continues to be economically affordable. Some service users perceived orthodox healthcare to be inexpensive due to the presence of national health insurance scheme. Exorbitant prices charged at TM clinics could be a hindrance to integration if policy makers do not have the political will or intention to include traditional health services in the national health insurance scheme [16, 38]. However, effective listening, trust, respect, sympathy, prompt delivery of services, effectiveness of therapy, receptiveness and effective communication between clients and TM practitioners could boost the integration process. Service users often evaluate satisfaction based on the effectiveness of therapy, responsiveness of service providers (efficient collaboration) and timely delivery of health services [56].

Participants had a high preference for TM integration into the formal health system. Similar results have been recounted in the works of Agyei-Baffour, Kudolo [10] and Boateng, Danso-Appiah [11] where service users preferred and were pleased with the practice of integrated health in the Ashanti region. The authors reported that service users felt safe patronising TM services and products from qualified and licensed TM practitioners [10]. In another study, community members demonstrated strong preference for integration by urging policy makers to intensify education to unify the two health systems as well as promote proper enforcement of policies governing the integration process [4]. Service users’ strong desire for collaboration between the two health systems could improve the process of integration by increasing consumers’ patronage of traditional health services at formalised health centres thereby eliminating consumer-led integration and promote safe TM practice [10]. Additionally, some demographic characteristics of participants were significantly associated with preference for TM integration in the regression analysis. For example, the present study has shown that people who earn less than GHC 500 are more likely to prefer TM integration than people who earn higher monthly incomes. The qualitative phase affirms this outcome as a similar trend was also observed. Results from both phases of the study clearly indicate low-income earners’ enthusiasm for the practice of integrated healthcare. These results however, contradict evidence from another study in Ghana where preference/usage of integrated services was reported to be largely associated with high-income earners [10]. We speculate that low-income earners’ preference for TM integration could be useful in expanding their knowledge about the practice of integrated health and improve their health-seeking behaviour. The use of the Food and Drug Authority reporting system on adverse drug reaction [57] serves as a monitoring system used in informing and improving the delivery of health services in Ghana particularly in the field of TM practice.

The study found that knowledge concerning TM integration is low among service users. Some participants were familiar with the licensing and/or regulations of TM practice in Ghana as well as training of TM practitioners at KNUST. However, the majority of participants were ignorant of the presence of TM clinics in selected public hospitals in the Ashanti region. This finding corroborates the results of studies conducted at one of the integrated health facility in the Ashanti region where clients reported not having knowledge of the presence of TM unit at the facility [10, 11]. This knowledge gap could be because of the absence of a written protocol or an agency to publicise the presence of TM departments in some government hospitals in Ghana [11, 16]. The non-existence of a protocol/agency might be an obstruction to the integration process because it could lead to ‘consumer-led’ integration and misconceptions about the system in its entirety, which can mar the quality of integrated healthcare practiced in Ghana [11, 38]. Our study suggests that sex, marital status, household size and residential status of participants were significantly associated with knowledge about TM integration. For example, findings from both quantitative and qualitative phases of our study show that males’ and urban settlers’ are more familiar with the practice of integrated healthcare in Ghana. This finding seems to support the pluralist model which postulates that gender and ethnicity/sub-groups should be core elements in defining and designing any future strategies regarding TM integration into national health systems to ensure greater involvement (cross-referral) and satisfaction for all stakeholders within the health system [58, 59].

Cross-referral is a tool needed to integrate TM effectively into formal health system (4, 38). However, study participants disclosed weak cross-referrals between the two health systems. Weak referral as reported in the current study is not surprising. A previous study in Ghana showed that orthodox health providers were only prepared to collaborate with TM practitioners on the condition that the Ministry of Health offers a well drafted document/blueprint describing roles and standard operating procedures, which apply to all stakeholders in the system [11, 60]. Likewise, referrals from TM practitioners to orthodox providers were also low. This could mean that to some TM practitioners, integration is a strategy to control TM practice by favouring those with a tertiary degree over those who practice within communities just as portrayed in a survey conducted in rural Nepal [61]. The minimal understanding and enthusiasm from both groups of health practitioners could make the integrated system unproductive hence produce unsatisfactory outcomes. This could impede the interaction between service users and synergistic teams of biomedical and traditional health providers.

The few service users who had interacted with the integrated system together with TM users narrated a satisfactory outcome. Satisfaction means that diagnosis, care or therapy achieves the preferred outcome from service users’ perspective [56]. The expectations of service users about the systems were met in this study. This means that quality of care attributes such as: conducive environment, empathy of practitioners as well as effectiveness of therapy were effectuated [56]. Satisfactory reports of clients might improve the Ghanaian integrated system through increased knowledge and patronage of integrated health services.

Generally, study participants regardless of their geographical location, demonstrated their support for TM integration. However, preferences have not yet migrated into engagement with the integrated system. The level of preference and tolerability from service users could facilitate successful practice of integrated health system in Ghana if manned properly. Countries such as China, Japan and Republic of Korea have successfully improved collaboration between the two health systems [14]. These Asian countries have emerged as the best health systems similar to standards in the developed world, due to effective absorption of traditional healthcare into mainstream health systems [14]. The success of these countries illustrates that with the availability of standardised knowledge, wide-ranging methodologies and rich scientific experiences in TM, the field could be a competent and efficient component of the Ghanaian health system. Therefore, study participants suggested that the Ghanaian integrated system could be enriched through proper processing, packaging and certification of TM products/services, professional training of TM providers as well as sensitisation of the service users through the media.

### Implication for practice

The Sustainable Development Goal (SDG) 3 focus on good health and wellbeing, ensuring a healthy life and promoting the wellbeing of all individuals irrespective of age [62]. In Ghana, TM has been greatly used by the population as first line of healthcare [12]. The role TM plays in health delivery in Ghana could serve as a significant basis for the attainment of this goal as well as lead to equitable access to health services in the country. The SDG 3 specifies that every individual can have access to high quality essential healthcare services at lower cost [62]. The attainment of the SDG 3 in Ghana could be thwarted because the study demonstrated that service users deemed approved TM products to be costly, thereby, affecting patronage of integrated health services. Actions necessary to improve integrated healthcare and consequently achievement of SDG 3 include:

- Willingness on the part of policy makers to include some of the approved TM products on the NHIS Drug List.
- Government legislative and regulatory instruments to increase the number of integrated health facilities in Ghana to promote patronage particularly in the rural areas where TM is widely used.
- A well-developed and documented partnership building strategy between all stakeholders particularly the health practitioners to strengthen cross referral between the two health systems.

### Strengths and limitation

The study presents relevant findings on healthcare accessibility, knowledge about integration and satisfaction derived from the various health systems in Ghana’s Ashanti region using a mixed methods approach. The existence of such knowledge is essential to inform and guide policy makers to modify existing policies, which might improve the implementation of the intervention. The selection of participants from both rural and urban districts increases the generalisability of the study results thereby making it useful for policy advice. However, participants could over-estimate or exaggerate their narrative given that TM use is prevalent in the study area.

## Conclusion

In conclusion, a considerable number of residents in Ashanti region patronise TM. The efficacious nature of TM and empathetic attitude of its practitioners show a positive indication or an enabler to the integration process. However, most of the participants were not aware of the presence of TM clinics in selected hospitals in the region, therefore, accounting for low level of engagement with the integrated system. Service users’ unfamiliarity with the presence of integrated health facilities in Ghana could be an impediment to the practice of integrated healthcare. Measures such as professional training of TM practitioners, proper processing, packaging and certification protocols, correct implementation approach and sensitisation of the public about the practice of an integrated system and its associated benefits (using a drafted protocol/authorised agency/media) could be helpful in refining the Ghanaian integrated system. If TM is integrated properly into the health system, it might create an opportunity for service users to access both TM and orthodox healthcare in a formalised setting, which is the highly preferred option on the part of service users. Likewise, the practice of integrated healthcare could guarantee the provision of quality healthcare for users since the system will draw on the strengths of both health systems to create an all-inclusive healthcare unit that will secure the safety and satisfaction of service users. Regular evaluation of patient satisfaction and outcome measures could be an effective strategy for improving health services delivery since evaluation is becoming an important component of health service design and implementation. With this in mind, there is the need for future studies to explore the perceptions and experiences of other stakeholders such as health practitioners and hospital administrators in relation to the practice of integrated health in Ghana.

## Supplementary Information

**Additional file 1.**

## Data Availability

The datasets used and / or analysed during the current study are available from the corresponding author on reasonable request.

## Abbreviations

AOR: Adjusted Odds Ratio
CI: Confidence Interval
COREQ: Consolidated Criteria for Reporting Qualitative Studies
FDA: Food and Drugs Authority
GHS: Ghana Cedis
KNUST: Kwame Nkrumah University of Science and Technology
NHIS: National Health Insurance Scheme
SPSS: Statistical Package for Social Sciences
TM: Traditional Medicine
USD: United States Dollar
WHO: World Health Organization

## References

[1] Osemene KP, Elujoba AA, Ilori MO (2011). A comparative assessment of herbal and orthodox medicines in Nigeria. Res J Med Sci.

[2] Sambo LG (2003). Integration of traditional medicine into health systems in the African region—The journey so far. Afr Health Monit..

[3] Abel C, Busia K (2005). An exploratory ethnobotanical study of the practice of herbal medicine by the Akan peoples of Ghana. Altern Med Rev.

[4] Gyasi RM, Poku AA, Boateng S, Amoah PA, Mumin AA, Obodai (2017). Integration for coexistence? Implementation of intercultural health care policy in Ghana from the perspective of service users and providers. J Integr Med.

[5] Astin JA (1998). Why patients use alternative medicine: results of a national study. J Am Med Assoc.

[6] Allam S, Moharam M, Alarfaj G (2014). Assessing patients’ preference for integrating herbal medicine within primary care services in Saudi Arabia. J Evid Based Complementary Altern Med.

[7] Lin V, Canaway R, Carter B (2015). Interface, interaction and integration: how people with chronic disease in Australia manage CAM and conventional medical services. Health Expect.

[8] Hollenberg D (2006). Uncharted ground: patterns of professional interaction among complementary/alternative and biomedical practitioners in integrative health care settings. Soc Sci Med.

[9] World Health Organization (2019). WHO Global Report onTraditional and Complementary Medicine 2019.

[10] Agyei-Baffour P, Kudolo A, Quansah DY, Boateng D (2017). Integrating herbal medicine into mainstream healthcare in Ghana: clients' acceptability, perceptions and disclosure of use. BMC Complement Altern Med.

[11] Boateng MA, Danso-Appiah A, Kofi Turkson B, Tersbøl BP (2016). Integrating biomedical and herbal medicine in Ghana -- experiences from the Kumasi South Hospital: a qualitative study. BMC Complement Altern Med.

[12] World Health Organization (2002). WHO traditional medicine strategy 2002-2005.

[13] Vasconi E, Owoahene-Acheampong S (2010). Recognition and integration of traditional medicine in Ghana: a perspective. Res Rev.

[14] World Health Organization (2000). Traditional and modern medicine: harmonizing the two approaches: a report of the consultation meeting on traditional and modern medicine: Harmonising the two approaches, 22-26 November 1999, Beijing, China.

[15] World Health Organization (2020). Traditional medicine in the WHO south east region: review of Progress 2014-2019.

[16] Appiah B, Amponsah IK, Poudyal A, Mensah MLK (2018). Identifying strengths and weaknesses of the integration of biomedical and herbal medicine units in Ghana using the WHO health systems framework: a qualitative study. BMC Complement Altern Med.

[17] Kretchy IA, Okere HA, Osafo J, Afrane B, Sarkodie J, Debrah P (2016). Perceptions of traditional, complementary and alternative medicine among conventional healthcare practitioners in Accra, Ghana: implications for integrative healthcare. J Integr Med.

[18] Aries MJ, Joosten H, Wegdam HH, van der Geest S (2007). Fracture treatment by bonesetters in Central Ghana: patients explain their choices and experiences. Tropical Med Int Health.

[19] Essegbey GO, Awuni S, Kraemer-Mbula E, Wunsch-Vincent S (2016). Herbal medicine in the informal sector of Ghana. The informal economy in developing nations: hidden engine of innovation? Intellectual property, innovation and economic development.

[20] James PB, Wardle J, Steel A, Adams J (2018). Traditional, complementary and alternative medicine use in sub-Saharan Africa: a systematic review. BMJ Glob.

[21] Yarney J, Donkor A, Opoku SY, Yarney L, Agyeman-Duah I, Abakah AC (2013). Characteristics of users and implications for the use of complementary and alternative medicine in Ghanaian cancer patients undergoing radiotherapy and chemotherapy: a cross-sectional study. BMC Complement Altern Med.

[22] Ampomah IG, Malau-Aduli BS, Malau-Aduli AEO, Emeto TI (2020). Effectiveness of integrated health systems in Africa: a systematic review. Medicina (Kaunas).

[23] Boadu AA, Asase A (2017). Documentation of herbal medicines used for the treatment and Management of Human Diseases by some communities in southern Ghana. Evid Based Complement Alternat Med.

[24] Wilmot D, Ameyaw EO, Amoako-Sakyi D, Boampong JN, Quashie NB (2017). In vivo efficacy of top five surveyed Ghanaian herbal anti-malarial products. Malar J.

[25] Buabeng KO, Duwiejua M, Dodoo AN, Matowe LK, Enlund H (2007). Self-reported use of anti-malarial drugs and health facility management of malaria in Ghana. Malar J.

[26] Affum AO, Shiloh DO, Adomako D (2013). Monitoring of arsenic levels in some ready-to-use anti-malaria herbal products from drug sales outlets in the Madina area of Accra, Ghana. Food Chem Toxicol.

[27] Bugyei KA, Boye GL, Addy ME (2010). Clinical efficacy of a tea-bag formulation of cryptolepis sanguinolenta root in the treatment of acute uncomplicated falciparum malaria. Ghana Med J.

[28] Peprah P, Agyemang-Duah W, Arthur-Holmes F, Budu HI, Abalo EM, Okwei R, Nyonyo J (2019). ‘We are nothing without herbs’: a story of herbal remedies use during pregnancy in rural Ghana. BMC complementary and alternative medicine..

[29] Oreagba IA, Oshikoya KA, Amachree M (2011). Herbal medicine use among urban residents in Lagos, Nigeria. BMC Complement Altern Med.

[30] Abt AB, Oh YJ, Huntington RA, Burkhart KK (1995). Chinese herbal medicine induced acute renal failure. Arch Intern Med.

[31] Addo VN (2007). Herbal medicines: socio-demographic characteristics and pattern of use by patients in a tertiary obstetrics and gynaecology unit. J Sci Technol.

[32] Nnorom IC, Osibanjo O, Eleke C (2006). Evaluation of human exposure to lead and calcium from some local Nigerian medicinal preparations. J Appl Sci.

[33] Obebi Cliff-Eribo K, Sammons H, Star K, Ralph Edwards I, Osakwe A, Choonara I (2016). Adverse drug reactions in Nigerian children: a retrospective review of reports submitted to the Nigerian Pharmacovigilance Centre from 2005 to 2012. Paediatr Int Child Health..

[34] Langlois-Klassen D, Kipp W, Jhangri GS, Rubaale T (2007). Use of traditional herbal medicine by AIDS patients in Kabarole District, Western Uganda. Am J Trop Med Hyg.

[35] Nordeng H, Bayne K, Havnen GC, Paulsen BS (2011). Use of herbal drugs during pregnancy among 600 Norwegian women in relation to concurrent use of conventional drugs and pregnancy outcome. Complement Ther Clin Pract.

[36] Campbell-Hall V, Petersen I, Bhana A, Mjadu S, Hosegood V, Flisher AJ (2010). Collaboration between traditional practitioners and primary health care staff in South Africa: developing a workable partnership for community mental health services. Transcult Psychiatry.

[37] Ghana Statistical Service (2013). 2010 population and housing census regional analytical reports: Ashanti_Region. Report. Ghana Statistical Service.

[38] Park YL, Canaway R (2019). Integrating traditional and complementary medicine with National Healthcare Systems for universal health coverage in Asia and the Western Pacific. Health Syst Reform.

[39] Ghana Statistical Service. 2010 population and housing census: summary report of final results. Ghana Statistical Service Accra; 2012.

[40] Ghana Statistical Service (2013). Population and housing census; analytical report, Offinso North District.

[41] GIS unit of Department of Geography and Regional Planning UCC, cartographer Map of showing study settlements in Kumasi metroplolis and Offinso north district. 2020.

[42] Kumasi Metrolpolitan Assembly, cartographer Kumasi Metropolitan Assembly (KMA) Health Facilities Map. 2015.

[43] Gyasi RM, Tagoe-Darko E, Mensah CM (2013). Use of traditional mediicne by HIV/AIDS patients in Kumasi metropolis, Ghana: a cross-sectional survey. Am Int J Complement Res.

[44] Creswell JW, Plano Clark VL (2011). The nature of mixed methods research. Designing and conducting mixed method research.

[45] Creswell PCVL (2018). Designing and conducting mixed methods research third edition ed.

[46] Creswell JW (2007). Five qualitative approaches to inquiry. Qualitative inquiry and research design: Choosing among five approaches.

[47] Greene JC, Caracelli JV, Graham WF (1989). Towards a conceptual framework for mixed-method evaluation designs. Educ Eval Policy Anal.

[48] Lwanga SK, Lemeshow S, Organization WH (1991). Sample size determination in health studies: a practical manual Geneva.

[49] Bellhouse DR (2005). Systematic sampling methods.

[50] Adjei B (2013). Utilisation of traditional herbal medicine and its role in health care delivery in Ghana: the case of Wassa Amenfi west district.

[51] Guest G, Bunce A, Johnson L (2006). How many interviews are enough?. Field Methods.

[52] O’Connor C, Joffe H (2020). Intercoder reliability in qualitative research: debates and practical guidelines. Int J Qual Methods..

[53] Tong A, Sainsbury P, Craig J (2007). Consolidated criteria for reporting qualitative research (COREQ): a 32-item checklist for interviews and focus groups. Int J Qual Health Care.

[54] Scharp KM, Thomas LJ (2019). Disrupting the humanities and social science binary: framing communication studies as a transformative discipline. Rev Commun.

[55] Mensah CM, Gyasi MR (2012). Use of herbal medicine in the management of malaria in the urban-periphery, Ghana. J Biol Agric Healthc.

[56] Mosadeghrad MA (2012). A conceptual framwork for quality of care. Mat Soc Med.

[57] Food and Drugs Authority (2016). Guidelines for Adverse Reaction Reporting.

[58] Lagro-Janssen T (2016). Sex, gender and health. Eur J Women's Stud.

[59] Ben-Arye E, Karkabi S, Shapira C, Schiff E, Lavie O, Keshet Y (2009). Complementary medicine in the primary care setting: results of a survey of gender and cultural patterns in Israel. Gend Med..

[60] Asante E, Avornyo R (2013). Enhancing healthcare system in Ghana through integration of traditional medicine. J Sociol Res..

[61] Poudyal AK, Jimba M, Murakami I, Silwal CR, Wakai S, Kuratsuji T (2003). A traditional healers’ training model in rural Nepal: strengthening their roles in community health. Tropical Med Int Health.

[62] Suzan G, Coulibaly SK (2018). Sustainable development goal #3, “health and well-being”, and the need for more integrative thinking. Vet Mex.

